# Locating Ultrasonic Signals Employing MEMS-On-Fiber Sensors

**DOI:** 10.3390/s19173696

**Published:** 2019-08-26

**Authors:** Wenrong Si, Chenzhao Fu, Haoyong Li, Jiaming Lv, Chaoyu Xiong, Peng Yuan, Yiting Yu

**Affiliations:** 1 State Grid Shanghai Electric Power Research Institute, Shanghai 200437, China; 2 Key Laboratory of Micro/Nano Systems for Aerospace (Ministry of Education), Northwestern Polytechnical University, Xi’an 710072, China; 3 Key Laboratory of Micro- and Nano-Electro-Mechanical Systems of Shaanxi Province, Northwestern Polytechnical University, Xi’an 710072, China; 4 Xi’an Maorong Power Equipment Co., Ltd., Xi’an 710048, China

**Keywords:** sound sensing, ultrasonic signal localization, MEMS-on-fiber sensor, time difference of arrival (TDOA)

## Abstract

Sound sensing finds wide applications in various fields, such as underwater detection, structural health monitoring, and medical diagnosis, to name just a few. Based on our previously developed MEMS-on-fiber sensors, showing the advantages of low cost, small volume, and high performance, a three-dimensional ultrasonic localization system employing four such sensors was established in this work. A time difference of arrival (TDOA) algorithm was utilized to analyze the acquired data and then calculate the accurate position of the ultrasonic signal source. Plenty of practical measurements were performed, and the derived localization deviation in the region of 2 m × 2 m × 1 m was about 2–5 mm. Outside this region, the deviation tended to increase due to the directional sensitivity existing in these sensors. As a result, for a more accurate localization requirement, more sensing probes are needed in order to depict a completely suitable application situation for MEMS technology.

## 1. Introduction

Ears are vitally important sensing organs for sound localization for human beings and animals. According to the difference in frequency, amplitude, and arrival time between the two ears, the capability of sound sensing is very convenient in everyday life. However, we can only hear sound in the frequency range between 20 Hz and 20 kHz. Various technologies and equipment have been developed to assist in sensing sounds covering a much wider range, from infrasonic sound [1] to ultrasonic sound [2,3,4], finding broad applications in fields such as underwater sensing [1], medical diagnosis [2], structural health monitoring [3], and indoor positioning [4].

Among the most commonly used technologies, piezoelectric transducers are quite popular, which can directly convert a sound signal into an electrical signal [5,6,7]. However, although they possess an excellent piezoelectric coefficient, piezoelectric thin films such as lead zirconate titanate (PZT) or organic polymers such as polyvinylidene fluoride (PVDF), lack the flexibility in the manufacturing process compared with the sensing film used for fiber-based sensors, since they can employ a much wider range of materials to ensure the features such as ultra-thin, ultra-flat, and ultra-smooth. Moreover, electrical transducers may fall into difficulties when installed in environments with complex electromagnetic fields, such as for monitoring the partial discharge in a power transformer. In these cases, fiber-based sensors show their superiority [3,8,9], which convert the sound signal into an optical signal and then simultaneously transmit inside, providing the advantages of not only immunity to electromagnetic interference (EMI), but also high sensitivity, compact size, and versatility. Moreover, in combining with the rapidly developing microelectromechanical system (MEMS) technology to define the thin micromechanical diaphragms, MEMS-on-fiber sensors [10,11,12,13] show great potential, due to their flexible control over the frequency response and sensitivity, as well as low cost and small size. 

In our previous research, we suggested a new configuration of MEMS-on-fiber sensors [14], for which the optical resonant cavity was directly comprised of a well-defined micromechanical reflecting membrane and the end surface of the fiber. The sensing mechanism, originating from the acoustic pressure’s mechanical deformation of the membrane, and subsequent induction of an intensity variation of light inside the fiber, was established. Because of the mature MEMS industry in controlling the geometrical parameters of the membrane—especially the thickness, as well as the relatively easy fabrication of the accurate spacers (which are important for constructing the optical resonant cavity)—the proposed MEMS-on-fiber sensors have demonstrated superior performance to most of their reported counterparts [15,16]. Furthermore, we investigated the directional sensitivity of these sensors due to the planar structure of the sensing membrane [15], showing that within the ±60° incident angle, the amplitude fluctuation is less than 5.9 dB. This property lays a solid foundation for applications such as the location of sound signals and ultrasonic navigation. In this paper, we establish a preliminary experimental platform for conceptual demonstration to locate the ultrasonic signals by employing four probes of our developed MEMS-on-fiber sensors. As a result, the ultrasonic signals in three-dimensional space could be oriented.

## 2. Experimental Preparation

### 2.1. Hardware System

**Figure 1:**
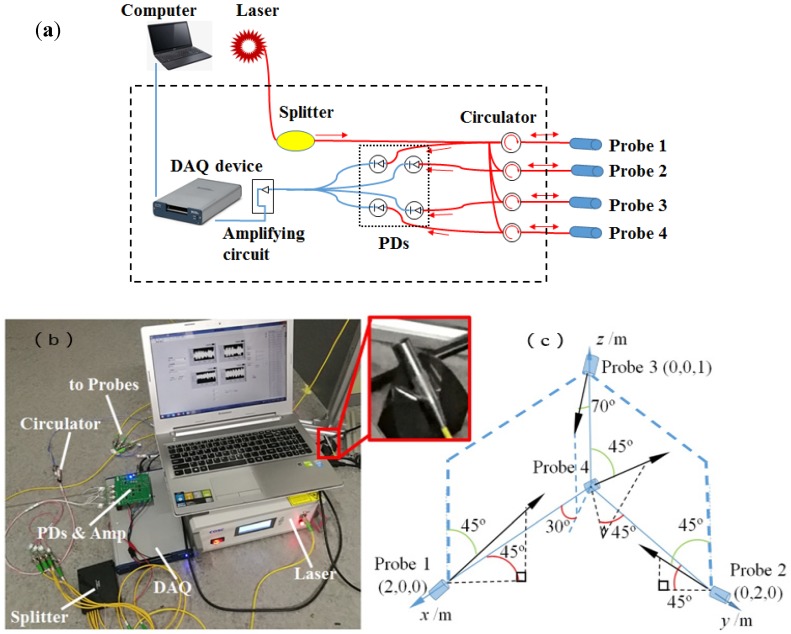
A schematic and experimental demonstration of the hardware system for locating ultrasonic signals. (**a**) The system configuration illustrating the key components of the whole system, including a distributed feedback (DFB) laser source, a one-to-five optical splitter, four fiber circulators connected to four MEMS-on-fiber sensors marked as Probes 1 to 4, photodiodes (PDs) for realizing the photon-to-electron conversion, as well as an amplifying circuit, a data-acquisition (DAQ) device, and a computer with specifically designed software to finish the data processing. (**b**) The experimental setup covering the above-mentioned key components with the enlarged inset showing the practically installed Probe 4. (**c**) A sketch diagram revealing the position relationship of all four sensing probes.

Figure 1 gives the schematic and experimental demonstration of the hardware system for locating the ultrasonic signals. As shown in Figure 1a, the whole system includes several key components, such as a distributed feedback (DFB, COSC, DFB-C-20) laser source (COSC Optoelectronics Co. Ltd., Beijing, China) with the wavelength of 1550 nm and specified power of 1 mW (long-term stability: ±0.05 dB at 8 h), an optical splitter (COSC Optoelectronics Co. Ltd., Beijing, China) to divide the original laser input into five equal parts, four fiber circulators (COSC Optoelectronics Co. Ltd., Beijing, China) behaving as both transmitter and receiver to/from four developed MEMS-on-fiber sensors (marked as Probe 1 to Probe 4), photodiode detectors (PDs; sensitivity: 0.9 A/W) for finishing the photon-to-electron conversion, a data-acquisition (DAQ, NI, USB-6341) device (National Instruments, Austin, TX, USA), and a computer (Lenovo, Beijing, China) with specifically designed software (Labview2013, National Instruments, Austin, TX, USA) to set up the DAQ and directly show the acquired data graphically. The practical components and connected system are demonstrated in Figure 1b, which also presents an enlarged view of the installed Probe 4. For all the sensing probes, the installation positions in this research can be seen in Figure 1c. Actually, the specific positions and directions of the probes are not unique, but will definitely influence the ultimate localization algorithm, as well as the localization accuracy that is related to the directional sensitivity of these sensors [15]. 

**Figure 2:**
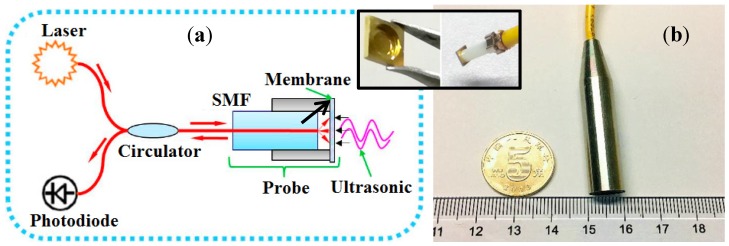
(**a**) Operational principle for detecting ultrasonic signals by utilizing the MEMS-on-fiber sensor. (**b**) Picture of a packaged sensing probe. SMF: single-mode fiber.

Figure 2a illustrates the operational principle for detecting ultrasonic signals by utilizing our MEMS-on-fiber sensors [16]. The DFB laser source with a narrow bandwidth of 0.1 nm is sent to the ultrasonic sensing probe through an optical circulator. The incident light transmits along the single-mode fiber (SMF) and comes into the Fabry–Perot (FP) cavity between the fiber’s end face and the attached micromechanical membrane covered with a gold film to enhance its reflectivity (as shown in the inset). Then, interference is generated between the reflected lights at the two cavity mirrors, whose intensity is modulated by the membrane vibration corresponding to the varying acoustic pressure, generated by a long lighter (the performance of the ultrasonic waves generated by the long lighter was detailed in our previous study) [15]. A photodiode collects the interfered light and converts it into electrical current. After the signal is amplified and filtered by a processing circuit, the voltage signal is then acquired by a DAQ device, and finally exported to the computer for further data processing by employing the time difference of arrival (TDOA) method. Figure 2b presents the picture of a packaged sensing probe. For more details, the readers are recommended to References [15,16].

### 2.2. Time Difference of Arrival (TDOA) Method

**Figure 3:**
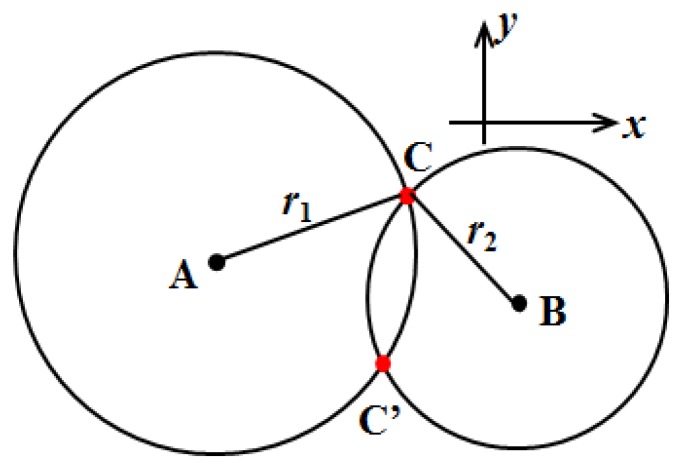
A schematic diagram to explain the time difference of arrival (TDOA) method.

In the domain of localization, the TDOA method is simple and widely adopted. It requires the measurement of the difference in time when the same signal arrives at two separate nodes. As shown in Figure 3, two nodes with the known positions A (*X_A_*, *Y_A_*) and B (*X_B_*, *Y_B_*) represent two sensing probes. The ultrasonic signal is generated at an unknown position, denoted as C (*X_C_*, *Y_C_*). Then, we can have the following equations:

(1) $$\{\begin{array}{c} {\left(X_{A}-X_{C}\right)}^{2}+{\left(Y_{A}-Y_{C}\right)}^{2}=r_{1}^{2}=v^{2}t_{1}^{2}\\ {\left(X_{B}-X_{C}\right)}^{2}+{\left(Y_{B}-Y_{C}\right)}^{2}=r_{2}^{2}=v^{2}t_{2}^{2}\\ r_{1}-r_{2}=△l=v\;\cdot △t=v\cdot (t_{1}-t_{2}) \end{array}$$

where *v* is the velocity of the sound in air. Considering the symmetrical distribution of the possible position of the ultrasonic signal (C and C′), another node (or sensing probe) with a known position is needed in order to obtain the definite position of the target. Similarly, four known nodes are necessary to localize the target in three-dimensional space.

**Figure 4:**
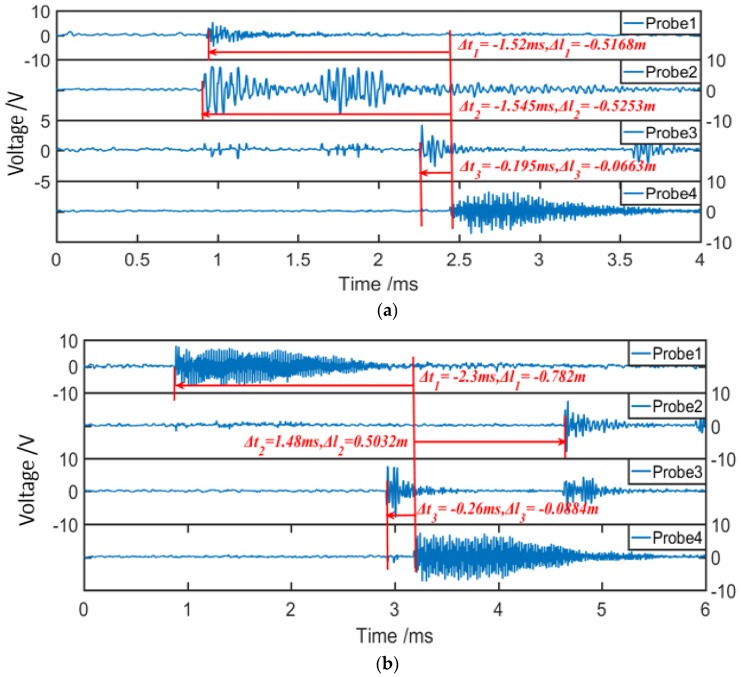
The time-domain responses of four MEMS-on-fiber sensors when the ultrasonic signal appears at the setting positions of (**a**) (1.5, 1.5, 0.5) and (**b**) (1.5, 0.5, 0.5).

According to the above analysis, taking our localization system in Figure 1c into account, two situations were designed, with the ultrasonic sources located at the positions of (1.5, 1.5, 0.5) and (1.5, 0.5, 0.5). Figure 4 gives the time-domain responses of four MEMS-on-fiber sensors for the two ultrasonic signals. Obviously, due to the different position relationships, the four probes detected the appearance of the same ultrasonic signal at different times. By utilizing this property, we could calculate the target position, and the results were (1.4980, 1.5051, 0.5174) and (1.4996, 0.5169, 0.5182), respectively, which were in good accordance with the ideal positions. 

## 3. Results and Discussion

**Figure 5:**
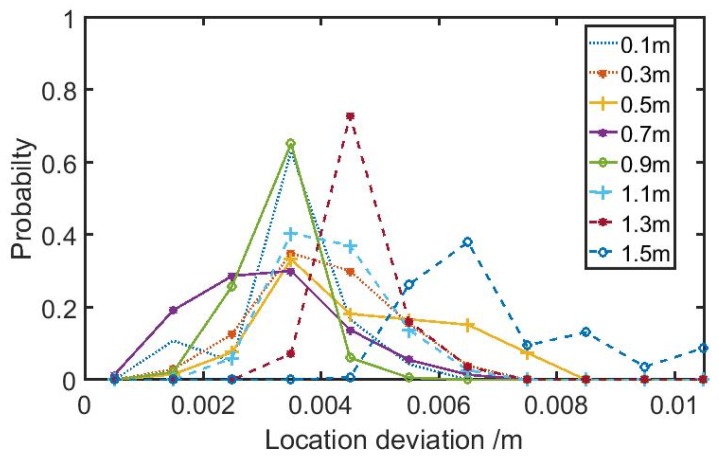
The localization deviation when the ultrasonic source was positioned at X = 1 m and Y = 1 m, yet with variable Z from 0.1 m to 1.5 m.

For more experimental verifications on the localization validity of our established system in this research, we performed various measurements when the ultrasonic source was located at different positions. Figure 5 presents the localization deviation when the ultrasonic source was positioned at X = 1 m and Y = 1 m, and changed Z from 0.1 m to 1.5 m with steps of 0.2 m. For each position, one hundred measurements were carried out. There was a general localization deviation of 2–5 mm. 

**Figure 6:**
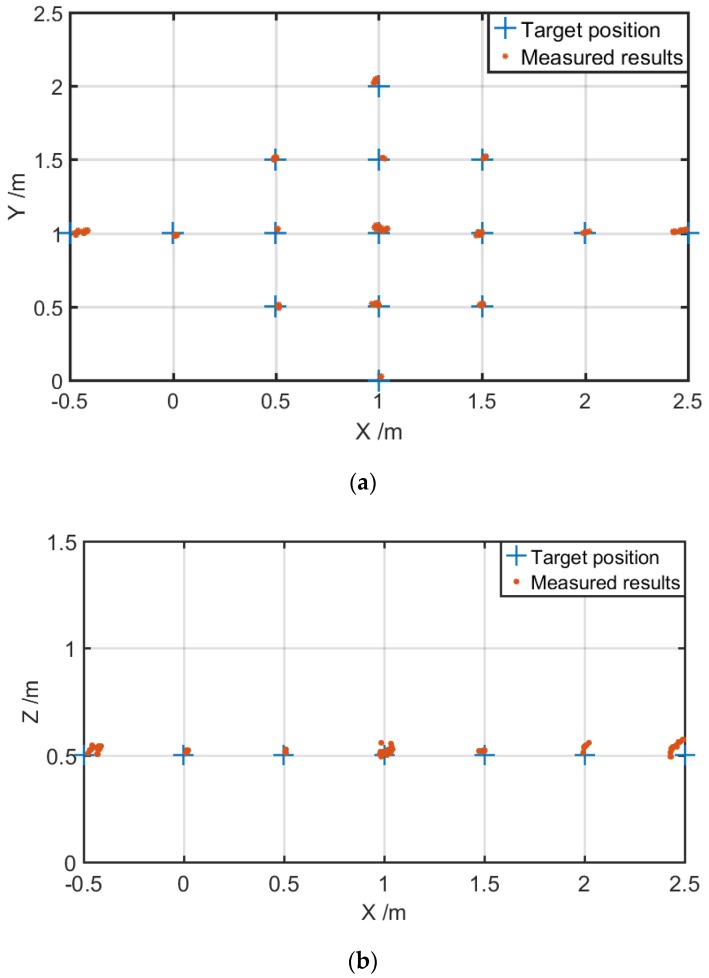

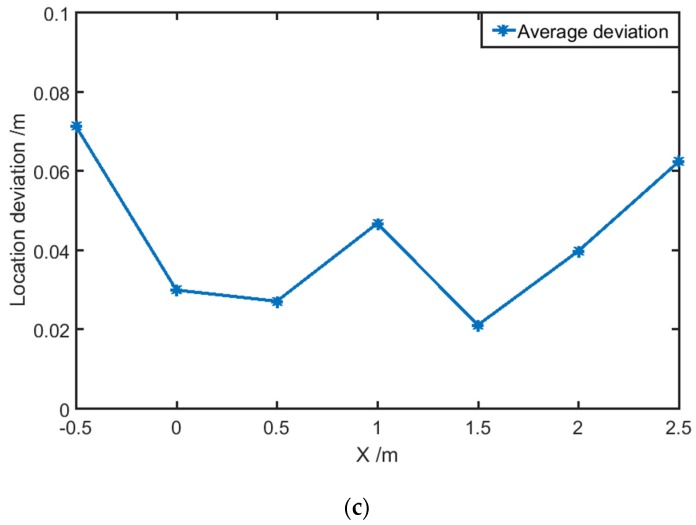
The localization results when the ultrasonic source was positioned at Z = 0.5 m, yet with variable X from −0.5 m to 2.5 m and Y from 0 m to 2 m. (**a**) The deviation projection in the XY plane; (**b**) The deviation projection in the XZ plane for Y = 1 m; and (**c**) the average localization deviation.

Then, a broader region with more target positions in space was investigated. For each position, twenty measurements were carried out. Figure 6 shows the localization results when the ultrasonic source was positioned at Z = 0.5 m and X was changed from −0.5 m to 2.5 m and Y from 0 m to 2 m, both with steps of 0.5 m. Figure 6a,b gives the deviation projection in the XY plane (Z = 0.5 m) and XZ plane (Y = 1 m), respectively. Figure 6c presents the average localization deviation for Figure 6b, from which we can see clearly that when the ultrasonic signal was positioned outside the distribution block of four probes, the deviation appeared to increase. This may be attributed to the directional sensitivity of these sensors. To further improve the localization accuracy, more probes should be introduced into the system to create a redundant data set of the target, and provide the possibility of selecting the most optimal four known probes to localize the ultrasonic target.

## 4. Conclusions

In this paper, a three-dimensional ultrasonic localization system based on four previously developed MEMS-on-fiber sensors was established. A TDOA algorithm was employed to analyze the acquired data and then calculate the accurate position of the ultrasonic signal source. According to a multitude of practical measurements, the localization deviation in the region of 2 m × 2 m × 1 m was about 2–5 mm. Outside this region, the deviation tended to increase. The main reason is that for ultrasonic sensors, whether piezoelectric type or membrane-on-fiber type, planar sensitive structures will definitely reveal a directional sensitivity. Therefore, for more accurate localization requirements, more sensing probes are needed. Fortunately, because of the MEMS processing technology for fabricating these sensors, they are a low-cost, small-volume, yet highly flexible method to define their responsive frequency and sensitivity. As a result, a thinner membrane will also help to improve the localization accuracy due to the enhanced sensitivity, as described in our previous reports. This kind of sound localization system shows potential for applications such as sound source seeking, malfunction detection, and even epicenter positioning.
